# Perspectives on the Potential Therapeutic Uses of Vesicles

**DOI:** 10.5772/57393

**Published:** 2013

**Authors:** Giovanni Camussi, Peter J. Quesenberry

**Affiliations:** 1 Department Medical Sciences, University of Torino, Torino, Italy; 2 Department of Medicine, the Warren Alpert Medical School of Brown University, Providence, RI, USA

**Keywords:** Exosomes, Microvesicles, Therapy

## Abstract

The role of extracellular vesicles as an important mediator of cell-to-cell communication has been well established by many studies that have shown their capability for exchanging proteins, bioactive lipids and nucleic acids. Extracellular vesicles have been implicated in several physiological and pathological processes according to the cell of origin. Identification of the innate properties of extracellular vesicles derived from stem cells and from immune cells has led to the possibility of their exploitation in regenerative medicine and immune therapies. As extracellular vesicles are able to cross biological barriers, express surface receptors and contain defined cargoes able to target specific cells/tissues, they may represent a biocompatible and effective tool for drug delivery. Herein, we review and discuss the perspectives related to the therapeutic opportunities of extracellular vesicles.

## 1. Introduction

Liposomes, which are synthetic phospholipid vesicles, have been used in the delivery of anticancer agents for the treatment of different solid tumours [1]. Anticancer agent-carrying liposomes are currently being investigated in several clinical trials [2-7]. In particular, polyethylene (PEG)-coated liposomes, which are long-lasting circulating liposomes, passively accumulate within tumours as a consequence of increased micro-vascular permeability and defective lymphatic drainage [8, 9]. To reduce the side effects of liposomes, targeting strategies have been developed using peptides, monoclonal antibodies and small organic molecules to achieve efficient internalization into the tumour vasculature and tumour cells [10]. Nevertheless, an ideal liposome, which specifically incorporates into target cells whilst avoiding the potential toxicity of its lipid membrane and the immunogenicity of targeting molecules, remains evasive. Naturally-occurring secreted vesicles, which are present in large amounts within biological fluids and therefore physiological constituents, could represent a valid alternative for overcoming some of the limitations posed by liposomes.

Secreted vesicles are heterogeneous populations of small vesicles released by eukaryotic cells.

They have been classified on the basis of the cell of origin, specific function, or of their biogenesis and are known in the literature by different names such as prostatosomes, cardiosomes, tolerosomes, microparticles, ectosomes, microvesicles and exosomes. Moreover, the cell-released vesicles also include apoptotic bodies, generated by blebbing of apoptotic cell membranes. Virtually all cells can secrete vesicles in basal conditions; however, this event is particularly evident for certain cell types and it may increase during cell proliferation and cell activation or after exposure to stress conditions [11]. The two major groups of non-apoptotic vesicles defined by their biogenesis are microvesicles and exosomes. Microvesicles have been defined as small vesicles generated by direct budding of the cell membrane, with a size ranging from 50 to 1000 nm. Microvesicles express surface receptors that vary according to the membrane composition of the cells of origin and may include molecules such as integrins, selectins and the CD40 ligand [12].

Exosomes have been defined as originating from inward budding of membranes of multivesicular bodies, followed by their fusion with the cell plasma membrane and release into the extra-cellular space [11, 13]. Exosomes, which are thought to be smaller than microvesicles (30-120 nm), express cell type-specific proteins and molecules that are considered specific markers of exosomes of different origin, such as CD63, CD9, CD81 tetraspanin family members, flotillin, CD82, Tsg101, Alix and other components of the endosomal sorting complex required for transport (ESCRT). Moreover, some exosomes may contain the heat shock 70kDa protein 8 and Rab-GTPases [13, 14].

This distinction, based on biogenesis, size, sedimentation on sucrose gradients, protein and lipid composition, remains confusing because the markers used for defining vesicles are frequently not exclusive, and may vary depending on the cell of origin. In addition, small vesicles have recently been reported to have a broad range and size [15, 16]. For this reason, the use of the generic term “extracellular vesicles (EV)” has been suggested for all secreted vesicles [17]. Recent studies have suggested that EV may act as vehicles for horizontal exchange of information between cells, independently from their biogenesis and characteristics [11-13, 18-20]. EV may either activate target cells by means of surface receptors or bioactive lipids, or by delivering their cargo, which may include transcription factors or nucleic acids, in particular, extra-cellular secreted RNA (exRNA) [21-24]. The exRNA that may convey paracrine/endocrine signals are present in all human biological fluids in degradative enzyme-protected forms, and are associated with protein carriers such as Ago2 and HDL or encapsulated within EV [25-29]. Both microvesicles and exosomes are exRNA enriched and include mRNA, microRNA (miRNA) and long non-coding RNA, and may enable transfer of genetic information between cells, which infers important physiological and pathological implications. The mRNA can be translated in the recipient cells, ensuing in the activation of intracellular pathways [22]. The miRNAs, which are known to regulate more than 80% of all protein-encoding genes and the long non-coding RNA (implicated in the regulation of the epigenome) may induce changes in the cell phenotype [30]. It is therefore conceivable that, under physiologic conditions, EV may play a critical role in signalling mechanisms for essential cellular and biological functions.

Naturally-occurring vesicles, given their properties of selectively targeting certain cell types or tissues in order to deliver their cargo, are potential candidates for therapeutic applications.

On one hand, the innate therapeutic potential of EV derived from certain cell types can be exploited, for example stem cells; on the other hand, EV may represent a biocompatible and effective tool for drug delivery, as they are able to cross biological barriers [31].

## 2. Innate therapeutic potential of EV

The possibility of exploiting the innate therapeutic potential of EV is based on the observation that, by delivering their bioactive cargo, EV plays a critical role in cell-to-cell cross talk [32]. EV derived from certain cell types may deliver information that reprogram target cells. This is the case, for example, of EV produced by stem/progenitor cells, which may convey information required for tissue regeneration or from immune modulatory cells that could potentially inhibit or promote specific immune responses.

### 2.1 Role of EV in stem/progenitor cell biology

Ratajczak et al. [21] demonstrated that EV released by murine embryonic stem cells may modulate hematopoietic progenitor phenotypes by transfer of proteins and mRNA, including Nanog, Rex-1, Oct-4 and HoxB4 early-transcription factors. EV from embryonic stem cells were also found to carry abundant miRNA, which can be transferred in vitro to mouse embryonic fibroblasts; this suggests that EV-derived stem cells can modulate the gene expression in recipient cells, as miRNAs regulate protein translation [33]. We found that EV released by endothelial progenitor cells (EPCs) activated angiogenesis in quiescent endothelial cells by transferring pro-angiogenic mRNA [22] and miRNA [34] fromEPCs to endothelial cells.

Adult human stem cells, such as bone marrow-derived multipotent stromal cells (MSC) and human liver stem cells (HLSC), secrete EV that contain specific subsets of functional mRNA [35, 36] and miRNA [24] associated with the mesenchymal phenotype, and control transcription, proliferation and immune regulation. EV released by MSC and HLSC contain the ribonucleoproteins TIA, TIAR, HuR and Staufen, which are responsible for RNA transport, stability and storage of mRNA, along with the Argonaute 2 (Ago2) protein, a critical component of the RNA-induced silencing complex (RISC), involved in the transport and maturation of miRNA [24]. By comparing the miRNA content of EV with that of parental cells, an enrichment of certain subsets of miRNA within EV was observed, suggesting non-random miRNA compartmentalization during EV formation [24]. This compartmentalization may be modulated by certain stimuli, as illustrated by EPC, where hypoxia was found to enhance EV expression of the angiomiR miR-126 and miR-296 [34].

Direct EV-mediated delivery of mRNA into bone marrow cells and induction of transcription was demonstrated by Aliotta et al. [37]. Several other studies using reporter mRNA have shown its translation into proteins following EV-mediated delivery, both in vitro and in vivo, indicating that transferred mRNA is functional [22, 24, 37]. Likewise, miRNAs transferred by EV were shown to block translation of target mRNA, suggesting that they may influence the phenotype of recipient cells [23, 24, 33].

Based on the properties of EV, Quesenberry et al. [38, 39] recently revised the hierarchical vision of the stem cell niche [40] by proposing an alternative model of stem cell biology defined as “continuum” and characterized by reversible change of stem cell phenotype during the cell cycle [41]. The terminal-differentiating stimulus is provided by environmental factors, including EV that may modulate the stem cell plasticity by means of genetic information exchange in a defined microenvironment. The genetic information exchange between tissue resident cells and stem cells is bi-directional and may account for stem cell phenotypic changes and activation of tissue regenerative programs. Transfer of lung-specific mRNAs, such as those coding for surfactant B and C and Clara cell-specific protein, to bone marrow cells via EV released from the injured lung cells was shown by Aliotta et al. [37]. Further work indicated that immediate expansion of lung-specific mRNA in marrow cells was due to transfer of both lung mRNA and lung-derived transcriptional regulation, but long-term genetic change was due to transcriptional modulation on epigenetic change in target marrow cells. [42]. Conversely, EV derived from stem/progenitor cells may modulate the phenotype of injured tissue and promote regeneration and cell differentiation. Therefore, the observation that the phenotype of target cells can be modified by EV-mediated transfer of exRNA provides a new perspective for the paracrine/endocrine hypothesis of stem cell action.

#### 2.1.1 EV derived from stem/progenitor cells in tissue regeneration

The beneficial effects of stem cell-based therapies are not supported by any tangible indication that in vivo stem cells are able to permanently engraft the injured tissues and substitute parenchymal cells, despite their in vitro plasticity. Instead, the current view is that stem cells induce regeneration by paracrine/endocrine mechanisms [43, 44]. This hypothesis is supported by a number of studies showing that stem cell-conditioned media hold the same beneficial properties of the stem cell of origin [45-47]. Moreover, a major contribution of resident cells in tissue regeneration after injury has been demonstrated in many organs such as the liver [48], heart [49] and kidneys [50].

In this context, if EV were able to reproduce the regenerative action of stem cells, they may provide an important new therapeutic perspective. We observed that EV from human bone marrow-derived MSCs were able to promote the recovery of acute kidney injury (AKI) in a manner similar to the cell of origin [35]. Moreover, EV administered with a therapeutic regimen in a lethal model of AKI induced significantly improved survival and tissue regeneration [51]. We have shown that MSC-derived EV obtained by differential centrifugations express several mesenchymal markers, including CD105, CD73, CD44 and CD29, as well as a LAMP-1 exosomal marker [51]. This healing effect of EV was associated with the transfer of human MSC-specific mRNA and its transient translation into proteins within the injured kidneys of SCID mice [35, 51]. Studies on bio-distribution indicated a selective accumulation within the injured kidney but not in the normal kidney. This tropism of EV for the injured tissue exploited the same adhesion molecules expressed by MSC. Within the kidney, EV are incorporated by injured endothelial and tubular cells, resulting in the prevention of cell apoptosis and the induction of proliferation of tubular epithelial cells, with a reconstitution of parenchymal integrity. In vitro, EV derived from human MSC were shown to induce a stem cell-like phenotype of renal tubular epithelial cells, with subsequent activation of regenerative programs [35].

The transcription regulators delivered by EV caused modification of gene expression in tubular epithelial cells, with a consequent up-regulation of the anti-apoptotic genes BIRC8, BCL-XL and BCL2 and down-regulation of the pro-apoptotic genes CASP1, CASP8 and LTA [51]. EV were also shown to transfer human IGF-1R mRNA from MSC to cisplatin-injured murine proximal tubular cells, thus enhancing tubular cell sensitivity to IGF-1, which is involved in renal regeneration [52]. This observation may provide an explanation for the powerful renoprotection provided by just a few MSC that are engrafted -onto the kidney in MSC-based therapy.

A renoprotective action has been described for EV derived from EPC in a model of ischemia-reperfusion injury (IRI), characterized by diffuse endothelial and tubular cell damage [34]. EPC-derived EV display pro-angiogenic properties, as they can transfer mRNA associated with the PI3K/AKT signalling pathway [22] and pro-angiogenic miR 126 and miR 296 [34]. Once injected into diseased animals, EV localize within peritubular capillaries and tubular cells, restraining tissue damage and favouring a rapid recovery from AKI, and preventing capillary rarefaction, glomerulosclerosis and tubulo-interstitial fibrosis, which causes chronic kidney damage [34].

Non-specific miRNA depletion of EV by Dicer knockdown in EPCs, specific depletion of miR-126 and miR-296 by siRNA transfection in EPC, or inhibition by the use of antagomirRs prevented the renoprotective effect of EV [3]. By means of a similar mechanism, EPC-derived EV were found to improve vascularization and favour muscle regeneration in a model of hind limb ischemia made by ligation and resection of the left femoral artery in SCID mice [53].

EV have also been shown to display a therapeutic effect in other organs. For example, in a murine model of myocardial ischemia/reperfusion injury, EV derived from the conditioned medium of MSC were shown to decrease infarction size [54]. Moreover, it was found that foetal tissue-derived MSC was able to produce elevated amounts of EV with cardio-protective activity suitable for therapeutic use [55]. In addition, EV released from cardiac progenitor cells may be exploited as a potential therapeutic resource for myocardial pathology [56].

Our group also showed that EV derived from HLSC were able to stimulate liver repair in 70% hepatectomized rats [36]. In a recent study, Li et al. [57] demonstrated that EV obtained from mesenchymal stem cells derived from the human umbilical cord alleviates liver fibrosis and protect hepatocytes in a carbon tetrachloride model of chronic liver injury.

A neuro-regenerative potential of MSC-derived EV has also recently been proposed [58]. Ischemic brain extracts enhance the expression of Mir 133b in MSC-derived EV, which has been shown to play a critical role in functional recovery after spinal cord injury in zebra fish [59].

Iglesias et al. [60] suggested a possible application of EV derived from MSC of normal human subjects in the correction of a genetic disorder known as cystinosis. EV were found to transfer cystinosin protein and mRNA to human cystinotic cells and to reduce in vitro cystine accumulation.

#### 2.1.2 EV-mediated reprogramming of tumour cells

Although the physiological functions of EV in different tissues are mostly uncharacterized, it is emerging that they not only depend on the EV-carried molecules, but are also influenced by the functional and metabolic state of target cells. Indeed, the same EV may exhibit contrary effects depending on the different states of activation or inhibition of particular metabolic pathways in recipient cells. For instance, EV released from HLSC are able to accelerate liver regeneration [36] whilst inhibiting hepatoma growth, as HLSC-derived EV carry antitumor miRNA, which promote tumour regression [61]. These miRNA lacking in tumour cells are delivered by EV and then reprogram tumour cells to a more benign phenotype. Similarly, EV derived from bone marrow-MSCs are able to induce regression of different tumours by inhibiting cell cycle progression and inducing apoptosis [62]. However, as previously shown for MSC, the time of EV delivery is critical and MSC-derived EV may in fact enhance tumour engraftment by promoting neoangiogenesis [63], or may induce regression of an established tumour by favouring tumour cell apoptosis [62], depending on when they are administered. Recent studies conducted by the Quesenberry and Chatterjee groups indicate that vesicles derived from normal prostate cells can reverse the chemoresistance and anchorage-independent growth of malignant prostate cancer cells in vitro [64].

#### 2.1.3 EV-mediated modulation of the immune response

Raposo et al. [65] first demonstrated that B lymphocytes secrete antigen-presenting vesicles, which were shown to express peptide-bound class II major histocompatibility complex (MHC) able to induce a T cell response. Since then, a number of studies recently reviewed by Gutierrez-Vasquez et al. [66] confirmed the potential involvement of EV modulation in the immune response. A critical role has been suggested for EV-mediated exchange of information at the level of immune synapses, leading to the initiation of the immune response. This innate ability of EV to potentiate an immune response can be exploited for cancer immune therapy. Indeed, Zitvogel et al. [67] demonstrated the possibility of eradicating murine tumours using exosomes expressing class I and class II MHC molecules derived from dendritic cells pulsed with tumour peptides. Subsequent studies have demonstrated the enhancement of the T cell response, the protection of T cells from apoptosis, the enhancement of the production of pro-inflammatory cytokines and natural killer activity [68]. Clinical trials based on vaccination with tumour antigen-loaded dendritic cell-derived exosomes are presently underway (ClinicalTrials.gov). On the other hand, vesicles derived from cells that possess immune modulatory properties, such as MSC [69] or IL10-treated dendritic cells [70] may display anti-inflammatory and immune inhibitory properties, which could be exploited in the treatment of immune-mediated diseases. It has been proposed that EV released from MSC may produce tolerogenic signals by stimulating production of IL10 and TGF beta anti-inflammatory cytokine, and by expansion of CD4+, CD25+ and Foxp3+ regulatory T cells [71].

## 3. Therapeutic potential of EV for drug delivery

Based on the knowledge that EV express surface receptors and contain selected patterns of proteins and exRNA, we can potentially generate EV expressing or containing desired molecules by engineering the cells of origin. The feasibility of this approach has been demonstrated by the Gould group through protein targeting EV by plasma-membrane anchors [72]. Moreover, miRNA or siRNA transfected within the cell of origin have been shown to result in their incorporation in secreted EV [24, 61]. Alvarez-Erviti et al. [73] generated EV capable of specifically delivering siRNA to oligodendrocytes, microglia and neurons in the brain of intravenously injected mice, by inducing dendritic cells to express the exosomal membrane protein Lamp2b fused to the neuron-specific RVG peptide. The therapeutic potential of this strategy was shown by the knockdown of BACE1 mRNA and protein, a therapeutic target in Alzheimer's disease [73]. This study also demonstrates that targeted EV can cross the blood-brain barrier and target neurons without significant immunogenicity or toxicity. It has also been shown by Zhuang et al. [74] that EV loaded with anti-inflammatory drugs, unlike liposomes, were able to cross the blood-brain barrier after intranasal administration. The therapeutic potential of this strategy was investigated in neuro-inflammation induced by lipopolysaccharide and in experimental encephalomyelitis with curcumin-containing EV. In addition, in a mouse model of glioblastoma, EV complexed with a stat3 inhibitor were used. To improve neuron targeting of EV, Andaloussi et al. [75] generated exosomes expressing the RVG peptide.

Another example of the use of EV in therapy was demonstrated by Ohno et al. [76], who engineered cells to obtain EV expressing the GE11 peptide fused with the transmembrane domain of platelet-derived growth factor receptor and which showed an efficient in vivo delivery of miRNA to breast cancer cells bearing the epidermal growth factor receptor (EGFR). Moreover, Akao et al. [77] demonstrated that after transfection of miR-143BPs in human monocytic leukaemia THP-1 cells, RNA was secreted within EV. After intravenous injection of shed EV containing miR-143BPs, the level of this miRNA was significantly increased in the serum, tumour and kidneys of the host animals [77]. These studies indicate that the ex vivo manipulation of EV donor cells may modify the miRNA content of EV; this could be an efficient strategy for delivering specific subsets of miRNA to target cells. Similarly, Van den Boorn et al. [78] used loaded exosomes to efficiently deliver siRNAs to target cells in vivo in mice.

Taken together, these experiments provide proof of the concept that EV represent a potential biocompatible vehicle for different therapeutic molecules, enhancing their stability, limiting their potential toxicity and immunogenicity, targeting specific cells/tissues and enabling them to cross biological barriers.

## 4. Clinical translation of EV-mediated therapies

Despite the promising results demonstrated in experimental animals and the preliminary clinical trials that have tested exosomes in the field of cancer (clinicaltrials.gov), several points should be elucidated before envisaging the use of either the innate therapeutic potential or engineered EV in clinics.

Firstly, there is an urgent requirement to develop large-scale methods for EV preparation. This implies identification of the best suitable cellular sources and culture conditions for GMP production of EV. Culture conditions are critical, as it is known that they may influence the yield and the content of EV and thus their bioactivity. The use of EV for regenerative medicine and for drug delivery needs a source of cells that are expandable in GMP conditions and that generate nonimmunogenic EV. MSC derived from bone marrow, fat or the umbilical cord are potential candidates, as they retain the immune-modulatory properties of MSC. However, their senescence after repeated culture passages may limit their use. To overcome this limitation, Chen et al. [79] immortalized human embryonic stem cell-derived MSCs, enabling large-scale production of exosomes. These MSC remained unchanged in quantity and quality, and the immortalizing oncogene was not detected within exosomes. However, cell immortalization may present safety concerns and cannot be accepted by regulatory agencies. Another potential source of EV are HLSC [36]. HLCS can be expanded on a large scale in GMP conditions; they do not undergo senescence and they maintain a stable karyotype up to the 24^th^ passage. We have found that these cells do not require immortalization and the derived EV share several properties with those of MSC.

Another critical point is to develop an easy, reproducible and efficient GMP purification protocol.

The gold standard protocol for EV purification against which all other techniques should be evaluated is based on differential ultracentrifugation to remove cell debris and large vesicles, and to collect the small vesicles. As the ultracentrifugation technique is time-consuming with a low yield and poses concerns about the loss of the biological activities of EV, some alternative techniques have been under evaluation, including immunoaffinity [80, 81] and ultrafiltration using membranes with different-sized pores, combined with gel filtration by liquid chromatography [55]. So far, no ideal scalable purification technique applicable to a GMP condition is available. EV purified by all these techniques contain heterogeneous populations and a more precise purification, by combining different methods and using a sucrose gradient, is not feasible for a GMP production. Therefore, it is critical to define the healing EV population and to understand how far purification should be taken. Questions that need to be answered are: would non-healing populations interfere with the desired biological activity? What is the purity level that is required? Moreover, a test of potency should be developed for comparison of different EV batches. Ideally, the test should be in vitro, easily reproducible and straightforward, and should be appropriately designed for each field of application. Finally, it is important to define identity marker/s that correlate with the EV potency. Pre-clinical studies are also needed to determine in vivo bio-distribution of labelled EV to evaluate whether EV localize in the required organs. Finally, the biosafety of acute and chronic administration of EV requires further studies.

## 5. Conclusions

EV have emerged as an important vehicle of information between cells, as they can transfer bioactive proteins, lipids and nucleic acids. This property can be exploited for therapy in different fields, as EV retain several biological activities of the cell of origin. EV released in physiological conditions have innate therapeutic potential. Stem cell-derived EV mimic the favourable effects of the cell of origin, thus promoting repair and limiting injury in several organs, as they are able to activate regenerative programs and coordinate tissue self-repair. Due to the expression of membrane receptors derived from the stem cell of origin, EV can localize at the site of injury and deliver their cargo to damaged cells. The complex constituent array of EV allow them to influence multiple cellular pathways involved in different pathological conditions. EV derived from immune cells may potentiate the immune response and can therefore be used in cancer therapy. The identification of molecules responsible for the biological effect of EV may provide critical information for engineering EV for therapeutic purposes. The possibility of developing specifically targeted and drug-loaded EV may allow for the development of new therapeutic strategies. Additional investigations into the pathological conditions that may benefit from an EV-based therapy, as well as a definition of suitable, scalable GMP protocols of EV production are needed. Moreover, the biosafety and pharmacokinetics of EV require further studies.

## References

[1] Schiffelers RM, Storm G (2008). Liposomal nanomedicines as anticancer therapeutics: beyond targeting tumor cells. Int J Pharm.

[2] Main C, Bojke L, Griffin S, Norman G, Barbieri M, Mather L, Stark D, Palmer S, Riemsma R (2006). Topotecan, pegylated liposomal doxorubicin hydrochloride and paclitaxel for second-line or subsequent treatment of advanced ovarian cancer: a systematic review and economic evaluation. Health Technol Assess.

[3] Ulrich-Pur H, Kornek GV, Haider K, Kwasny W, Payrits T, Dworan N, Vormittag L, Depisch D, Lang F, Scheithauer W (2007). Phase II trial of pegylated liposomal doxorubicin (Caelyx) plus gemcitabine in chemotherapeutically pretreated patients with advanced breast cancer. Acta Oncol.

[4] Wagner S, Peters O, Fels C, Janssen G, Liebeskind AK, Sauerbrey A, Suttorp M, Hau P, Wolff JE (2008). Pegylated-liposomal doxorubicin and oral topotecan in eight children with relapsed high-grade malignant brain tumors. J Neurooncol.

[5] Piguet AC, Semela D, Keogh A, Wilkens L, Stroka D, Stoupis C, St-Pierre MV, Dufour JF (2008). Inhibition of mTOR in combination with doxorubicin in an experimental model of hepatocellular carcinoma. J Hepatol.

[6] Northfelt DW, Martin FJ, Working P, Volberding PA, Russell J, Newman M, Amantea MA, Kaplan LD (1996). Doxorubicin encapsulated in liposomes containing surface-bound polyethylene glycol: pharmacokinetics, tumor localization, and safety in patients with AIDS-related Kaposi's sarcoma. J Clin Pharmacol.

[7] Allen TM (1994). Long-circulating (sterically stabilized) liposomes for targeted drug delivery. Trends Pharmacol Sci.

[8] Maeda H, Wu J, Sawa T, Matsumura Y, Hori K (2000). Tumor vascular permeability and the EPR effect in macromolecular therapeutics: a review. J Control Release.

[9] Greish K (2007). Enhanced permeability and retention of macromolecular drugs in solid tumors: a royal gate for targeted anticancer nanomedicines. J Drug Target.

[10] Grange C, Geninatti-Crich S, Esposito G, Alberti D, Tei L, Bussolati B, Aime S, Camussi G (2010). Combined delivery and magnetic resonance imaging of neural cell adhesion molecule-targeted doxorubicin-containing liposomes in experimentally induced Kaposi's sarcoma. Cancer Res.

[11] Cocucci E, Racchetti G, Meldolesi J (2009). Shedding microvesicles: artefacts no more. Trends Cell Biol.

[12] Lee Y, El Andaloussi S, Wood MJ (2012). Exosomes and microvesicles: extracellular vesicles for genetic information transfer and gene therapy. Hum Mol Genet.

[13] Mathivanan S, Ji H, Simpson RJ (2010). Exosomes: extracellular organelles important in intercellular communication. J Proteomics.

[14] Mathivanan S, Fahner CJ, Reid GE, Simpson RJ (2012). ExoCarta 2012: database of exosomal proteins, RNA and lipids. Nucleic Acids Res.

[15] Fang Y, Wu N, Gan X, Yan W, Morrell JC, Gould SJ (2007). Higher-order oligomerization targets plasma membrane proteins and HIV gag to exosomes. PLoS Biol.

[16] Dragovic RA, Gardiner C, Brooks AS, Tannetta DS, Ferguson DJ, Hole P, Carr B, Redman CW, Harris AL, Dobson PJ, Harrison P, Sargent IL (2011). Sizing and phenotyping of cellular vesicles using Nanoparticle Tracking Analysis. Nanomedicine.

[17] Gould SJ, Raposo G (2013). As we wait: coping with an imperfect nomenclature for extracellular vesicles. Journal of extracellular vesicles.

[18] Ratajczak J, Wysoczynski M, Hayek F, JanowskaWieczorek A, Ratajczak MZ (2006). Membrane-derived microvesicles: important and underappreciated mediators of cell-to-cell communication. Leukemia.

[19] Théry C, Ostrowski M, Segura E (2009). Membrane vesicles as conveyors of immune responses. Nat Rev Immunol.

[20] György B, Szabó TG, Pásztói M, Pál Z, Misják P, Aradi B, László V, Pállinger E, Pap E, Kittel A, Nagy G, Falus A, Buzás EI (2011). Membrane vesicles, current state-of-the-art: emerging role of extracellular vesicles. Cell Mol Life Sci.

[21] Ratajczak J, Miekus K, Kucia M, Zhang J, Reca R, Dvorak P, Ratajczak MZ (2006). Embryonic stem cell-derived microvesicles reprogram hematopoietic progenitors: evidence for horizontal transfer of mRNA and protein delivery. Leukemia.

[22] Deregibus MC, Cantaluppi V, Calogero R, Lo Iacono M, Tetta C, Biancone L, Bruno S, Bussolati B, Camussi G (2007). Endothelial progenitor cell derived microvesicles activate an angiogenic program in endothelial cells by a horizontal transfer of mRNA. Blood.

[23] Valadi H, Ekström K, Bossios A, Sjöstrand M, Lee JJ, Lötvall JO (2007). Exosome-mediated transfer of mRNAs and microRNAs is a novel mechanism of genetic exchange between cells. Nat Cell Biol.

[24] Collino F, Deregibus MC, Bruno S, Sterpone L, Aghemo G, Viltono L, Tetta C, Camussi G (2010). Microvesicles derived from adult human bone marrow and tissue specific mesenchymal stem cells shuttle selected pattern of miRNAs. PLoS One.

[25] Chen X, Liang H, Zhang J, Zen K, Zhang CY (2012). Secreted microRNAs: a new form of intercellular communication. Trends Cell Biol.

[26] Gallo A, Tandon M, Alevizos I, Illei GG (2012). The majority of microRNAs detectable in serum and saliva is concentrated in exosomes. PLoS One.

[27] Wang K, Zhang S, Weber J, Baxter D, Galas DJ (2010). Export of microRNAs and microRNA-protective protein by mammalian cells. Nucleic Acids Res.

[28] Arroyo JD, Chevillet JR, Kroh EM, Ruf IK, Pritchard CC, Gibson DF, Mitchell PS, Bennett CF, PogosovaAgadjanyan EL, Stirewalt DL, Tait JF, Tewari M (2011). Argonaute2 complexes carry a population of circulating microRNAs independent of vesicles in human plasma. Proc Natl Acad Sci U S A.

[29] Vickers KC, Palmisano BT, Shoucri BM, Shamburek RD, Remaley AT (2011). MicroRNAs are transported in plasma and delivered to recipient cells by high-density lipoproteins. Nat Cell Biol.

[30] Whitehead J, Pandey GK, Kanduri C (2009). Regulation of the mammalian epigenome by long noncoding RNAs. Biochim Biophys Acta.

[31] Kooijmans SA, Vader P, van Dommelen SM, van Solinge WW, Schiffelers RM (2012). Exosome mimetics: a novel class of drug delivery systems. Int J Nanomedicine.

[32] EL Andaloussi S, Mäger I, Breakefield XO, Wood MJ (2013). Extracellular vesicles: biology and emerging therapeutic opportunities. Nat Rev Drug Discov.

[33] Yuan A, Farber EL, Rapoport AL, Tejada D, Deniskin R, Akhmedov NB, Farber DB (2009). Transfer of microRNAs by embryonic stem cell microvesicles. PLoS One.

[34] Cantaluppi V, Gatti S, Medica D, Figliolini F, Bruno S, Deregibus MC, Sordi A, Biancone L, Tetta C, Camussi G (2012). Microvesicles derived from endothelial progenitor cells protect the kidney from ischemia-reperfusion injury by microRNA-dependent reprogramming of resident renal cells. Kidney Int.

[35] Bruno S, Grange C, Deregibus MC, Calogero RA, Saviozzi S, Collino F, Morando L, Busca A, Falda M, Bussolati B, Tetta C, Camussi G (2009). Mesenchymal stem cell-derived microvesicles protect against acute tubular injury. J Am Soc Nephrol.

[36] Herrera MB, Fonsato V, Gatti S, Deregibus MC, Sordi A, Cantarella D, Calogero R, Bussolati B, Tetta C, Camussi G (2010). Human liver stem cell-derived microvesicles accelerate hepatic regeneration in hepatectomized rats. J Cell Mol Med.

[37] Aliotta JM, Pereira M, Johnson KW, de Paz N, Dooner MS, Puente N, Ayala C, Brilliant K, Berz D, Lee D, Ramratnam B, McMillan PN, Hixson DC, Josic D, Quesenberry PJ (2010). Microvesicle entry into marrow cells mediates tissue-specific changes in mRNA by direct delivery of mRNA and induction of transcription. Exp Hematol.

[38] Quesenberry PJ (2006). The continuum model of marrow stem cell regulation. Curr Opin Hematol.

[39] Quesenberry P, Abedi M, Dooner M, Colvin G, Sanchez-Guijo FM, Aliotta J, Pimentel J, Dooner G, Greer D, Demers D, Keaney P, Peterson A, Luo L, Foster B (2005). The marrow cell continuum: stochastic determinism. Folia Histochem Cytobiol.

[40] Till JE, McCulloch EA, Siminovitch L (1964). (1964) A stochastic model of stem cell proliferation, based on the growth of spleen colony-forming cells. Proc Natl Acad Sci U S A.

[41] Quesenberry PJ, Aliotta JM (2008). The paradoxical dynamism of marrow stem cells: considerations of stem cells, niches, and microvesicles. Stem Cell Rev.

[42] Aliotta JM, Pereira M, Li M, Amaral A, Sorokina A, Dooner MS, Sears EH, Brilliant K, Ramratnam B, Hixson DC, Quesenberry P (2012). Stable cell fate changes in marrow cells induced by lung-derived vesicles. J Extracellular Vesicles.

[43] Ratajczak MZ, Kucia M, Jadczyk T, Greco NJ, Wojakowski W, Tendera M, Ratajczak J (2012). Pivotal role of paracrine effects in stem cell therapies in regenerative medicine: can we translate stem cell-secreted paracrine factors and microvesicles into better therapeutic strategies?. Leukemia.

[44] Gnecchi M, Danieli P, Cervio E (2012). Mesenchymal stem cell therapy for heart disease. Vascul Pharmacol.

[45] Gnecchi M, He H, Noiseux N, Liang OD, Zhang L, Morello F, Mu H, Melo LG, Pratt RE, Ingwall JS, Dzau VJ (2006). Evidence supporting paracrine hypothesis for Akt-modified mesenchymal stem cell-mediated cardiac protection and functional improvement. FASEB J.

[46] Timmers L, Lim SK, Arslan F, Armstrong JS, Hoefer IE, Doevendans PA, Piek JJ, El Oakley RM, Choo A, Lee CN, Pasterkamp G, de Kleijn DP (2007). Reduction of myocardial infarct size by human mesenchymal stem cell conditioned medium. Stem Cell Res.

[47] Bi B, Schmitt R, Israilova M, Nishio H, Cantley LG (2007). Stromal cells protect against acute tubular injury via an endocrine effect. J Am Soc Nephrol.

[48] Best DH, Coleman WB (2011). Activation and regulation of reserve liver progenitor cells. Vitam Horm.

[49] Torella D, Ellison GM, Mendez-Ferrer S, Ibanez B, Nadal-Ginard B (2006). Resident human cardiac stem cells: role in cardiac cellular homeostasis and potential for myocardial regeneration. Nat Clin Pract Cardiovasc Med.

[50] Ishibe S, Cantley LG (2008). (2008) Epithelial-mesenchymal-epithelial cycling in kidney repair. Curr Opin Nephrol Hypertens.

[51] Bruno S, Grange C, Collino F, Deregibus MC, Cantaluppi V, Biancone L, Tetta C, Camussi G (2012). Microvesicles derived from mesenchymal stem cells enhance survival in a lethal model of acute kidney injury. PLoS One.

[52] Tomasoni S, Longaretti L, Rota C, Morigi M, Conti S, Gotti E, Capelli C, Introna M, Remuzzi G, Benigni A (2013). Transfer of growth factor receptor mRNA via exosomes unravels the regenerative effect of mesenchymal stem cells. Stem Cells Dev.

[53] Ranghino A, Cantaluppi V, Grange C, Vitillo L, Fop F, Biancone L, Deregibus MC, Tetta C, Segoloni GP, Camussi G (2012). Endothelial progenitor cell-derived microvesicles improve neovascularization in a murine model of hind limb ischemia. Int J Immunopathol Pharmacol.

[54] Lai RC, Arslan F, Lee MM, Sze NS, Choo A, Chen TS, Salto-Tellez M, Timmers L, Lee CN, El Oakley RM, Pasterkamp G, de Kleijn DP, Lim SK (2010). Exosome secreted by MSC reduces myocardial ischemia/reperfusion injury. Stem Cell Res.

[55] Lai RC, Arslan F, Tan SS, Tan B, Choo A, Lee MM, Chen TS, Teh BJ, Eng JK, Sidik H, Tanavde V, Hwang WS, Lee CN, El Oakley RM, Pasterkamp G, de Kleijn DP, Tan KH, Lim SK (2010). Derivation and characterization of human fetal MSCs: an alternative cell source for large-scale production of cardioprotective microparticles. J Mol Cell Cardiol.

[56] Chen L, Wang Y, Pan Y, Zhang L, Shen C, Qin G, Ashraf M, Weintraub N, Ma G, Tang Y (2013). Cardiac progenitor-derived exosomes protect ischemic myocardium from acute ischemia/reperfusion injury. Biochem Biophys Res Commun.

[57] Li T, Yan Y, Wang B, Qian H, Zhang X, Shen L, Wang M, Zhou Y, Zhu W, Li W, Xu W (2013). Exosomes derived from human umbilical cord mesenchymal stem cells alleviate liver fibrosis. Stem Cells Dev.

[58] Xin H, Li Y, Buller B, Katakowski M, Zhang Y, Wang X, Shang X, Zhang ZG, Chopp M (2012). Exosome-mediated transfer of miR-133b from multipotent mesenchymal stromal cells to neural cells contributes to neurite outgrowth. Stem Cells.

[59] Yu YM, Gibbs KM, Davila J, Campbell N, Sung S, Todorova TI, Otsuka S, Sabaawy HE, Hart RP, Schachner M (2011). MicroRNA miR-133b is essential for functional recovery after spinal cord injury in adult zebrafish. Eur J Neurosci.

[60] Iglesias DM, El-Kares R, Taranta A, Bellomo F, Emma F, Besouw M, Levtchenko E, Toelen J, van den Heuvel L, Chu L, Zhao J, Young YK, Eliopoulos N, Goodyer P (2012). Stem cell microvesicles transfer cystinosin to human cystinotic cells and reduce cystine accumulation in vitro. PLoS One.

[61] Fonsato V, Collino F, Herrera MB, Cavallari C, Deregibus MC, Cisterna B, Bruno S, Romagnoli R, Salizzoni M, Tetta C, Camussi G (2012). Human liver stem cell-derived microvesicles inhibit hepatoma growth in SCID mice by delivering antitumor microRNAs. Stem Cells.

[62] Bruno S, Collino F, Deregibus MC, Grange C, Tetta C, Camussi G (2013). Microvesicles derived from human bone marrow mesenchymal stem cells inhibit tumor growth. Stem Cells Dev.

[63] Zhu W, Huang L, Li Y, Zhang X, Gu J, Yan Y, Xu X, Wang M, Qian H, Xu W (2012). Exosomes derived from human bone marrow mesenchymal stem cells promote tumor growth in vivo. Cancer Lett.

[64] Panagopoulos K, DelTatto M, Pantazatos D, Mills DR, Renzulli J, Quesenberry P, Chatterjee D (2013). Reversal of chemosensitivity and induction of cell malignancy of a non-malignant prostate cancer cell line upon extracellular vesicle exposure. J Extracellular Vesicles.

[65] Raposo G, Nijman HW, Stoorvogel W, Liejendekker R, Harding CV, Melief CJ, Geuze HJ (1996). B lymphocytes secrete antigen-presenting vesicles. J Exp Med.

[66] Gutiérrez-Vázquez C, Villarroya-Beltri C, Mittelbrunn M, Sánchez-Madrid F (2013). Transfer of extracellular vesicles during immune cell-cell interactions. Immunol Rev.

[67] Zitvogel L, Regnault A, Lozier A, Wolfers J, Flament C, Tenza D, Ricciardi-Castagnoli P, Raposo G, Amigorena S (1998). Eradication of established murine tumors using a novel cell-free vaccine: dendritic cell-derived exosomes. Nat Med.

[68] Chaput N, Théry C (2011). Exosomes: immune properties and potential clinical implementations. Semin Immunopathol.

[69] English K (2013). Mechanisms of mesenchymal stromal cell immunomodulation. Immunol Cell Biol.

[70] Kim SH, Lechman ER, Bianco N, Menon R, Keravala A, Nash J, Mi Z, Watkins SC, Gambotto A, Robbins PD (2005). Exosomes derived from IL-10-treated dendritic cells can suppress inflammation and collagen-induced arthritis. J Immunol.

[71] Mokarizadeh A, Delirezh N, Morshedi A, Mosayebi G, Farshid AA, Mardani K (2012). Microvesicles derived from mesenchymal stem cells: potent organelles for induction of tolerogenic signaling. Immunol Lett.

[72] Shen B, Wu N, Yang JM, Gould SJ (2011). Protein targeting to exosomes/microvesicles by plasma membrane anchors. J Biol Chem.

[73] Alvarez-Erviti L, Seow Y, Yin H, Betts C, Lakhal S, Wood MJ (2011). Delivery of siRNA to the mouse brain by systemic injection of targeted exosomes. Nat Biotechnol.

[74] Zhuang X, Xiang X, Grizzle W, Sun D, Zhang S, Axtell RC, Ju S, Mu J, Zhang L, Steinman L, Miller D, Zhang HG (2011). Treatment of brain inflammatory diseases by delivering exosomes encapsulated anti-inflammatory drugs from the nasal region to the brain. Mol Ther.

[75] El Andaloussi S, Lakhal S, Mäger I, Wood MJ (2013). Exosomes for targeted siRNA delivery across biological barriers. Adv Drug Deliv Rev.

[76] Ohno S, Takanashi M, Sudo K, Ueda S, Ishikawa A, Matsuyama N, Fujita K, Mizutani T, Ohgi T, Ochiya T, Gotoh N, Kuroda M (2013). Systemically injected exosomes targeted to EGFR deliver antitumor microRNA to breast cancer cells. Mol Ther.

[77] Akao Y, Iio A, Itoh T, Noguchi S, Itoh Y, Ohtsuki Y, Naoe T (2011). Microvesicle-mediated RNA molecule delivery system using monocytes/macrophages. Mol Ther.

[78] van den Boorn JG, Schlee M, Coch C, Hartmann G (2011). SiRNA delivery with exosomes nanoparticles. Nat Biotechnol.

[79] Chen TS, Arslan F, Yin Y, Tan SS, Lai RC, Choo AB, Padmanabhan J, Lee CN, de Kleijn DP, Lim SK (2011). Enabling a robust scalable manufacturing process for therapeutic exosomes through oncogenic immortalization of human ESC-derived MSCs. J Transl Med.

[80] Clayton A, Court J, Navabi H, Adams M, Mason MD, Hobot JA, Newman GR, Jasani B (2001). Analysis of antigen presenting cell derived exosomes, based on immuno-magnetic isolation and flow cytometry. J Immunol Methods.

[81] Mathivanan S, Lim JW, Tauro BJ, Ji H, Moritz RL, Simpson RJ (2010). Proteomics analysis of A33 immunoaffinity-purified exosomes released from the human colon tumor cell line LIM1215 reveals a tissue-specific protein signature. Mol Cell Proteomics.

